# Acetic Acid Activates the AMP-Activated Protein Kinase Signaling Pathway to Regulate Lipid Metabolism in Bovine Hepatocytes

**DOI:** 10.1371/journal.pone.0067880

**Published:** 2013-07-04

**Authors:** Xinwei Li, Hui Chen, Yuan Guan, Xiaobing Li, Liancheng Lei, Juxiong Liu, Liheng Yin, Guowen Liu, Zhe Wang

**Affiliations:** Key Laboratory of Zoonosis, Ministry of Education, College of Veterinary Medicine, Jilin University, Changchun, Jilin, China; Boston University School of Medicine, United States of America

## Abstract

The effect of acetic acid on hepatic lipid metabolism in ruminants differs significantly from that in monogastric animals. Therefore, the aim of this study was to investigate the regulation mechanism of acetic acid on the hepatic lipid metabolism in dairy cows. The AMP-activated protein kinase (AMPK) signaling pathway plays a key role in regulating hepatic lipid metabolism. *In vitro*, bovine hepatocytes were cultured and treated with different concentrations of sodium acetate (neutralized acetic acid) and BML-275 (an AMPKα inhibitor). Acetic acid consumed a large amount of ATP, resulting in an increase in AMPKα phosphorylation. The increase in AMPKα phosphorylation increased the expression and transcriptional activity of peroxisome proliferator-activated receptor α, which upregulated the expression of lipid oxidation genes, thereby increasing lipid oxidation in bovine hepatocytes. Furthermore, elevated AMPKα phosphorylation reduced the expression and transcriptional activity of the sterol regulatory element-binding protein 1c and the carbohydrate responsive element-binding protein, which reduced the expression of lipogenic genes, thereby decreasing lipid biosynthesis in bovine hepatocytes. In addition, activated AMPKα inhibited the activity of acetyl-CoA carboxylase. Consequently, the triglyceride content in the acetate-treated hepatocytes was significantly decreased. These results indicate that acetic acid activates the AMPKα signaling pathway to increase lipid oxidation and decrease lipid synthesis in bovine hepatocytes, thereby reducing liver fat accumulation in dairy cows.

## Introduction

The mechanism of carbohydrate digestion and nutrient metabolism in ruminants differs significantly from that in monogastric animals. Cellulose and starch are the main carbohydrates for ruminants. Ruminal micro-organisms convert cellulose and starch to volatile fatty acids (VFAs, including acetic acid, propionic acid, and butyric acid), carbon dioxide, and methane. VFAs are the main precursors of lipid and glucose synthesis in ruminants [1]. Acetic acid, which accounts for 70–75% of VFAs, is absorbed by the rumen wall and neutralized by conversion to acetate in the blood. The blood acetate concentration in dairy cows is 3.6 mM, which is dozens of times higher than that in monogastric animals [2]. The biological function of acetic acid in dairy cows is also different from that in humans and mice. Acetic acid is mainly used for milk fat synthesis in dairy cows [2]. However, in humans and mice, acetic acid is mainly used to generate energy through tricarboxylic acid cycle in hepatocytes [3]. In recent years, it has become evident that acetic acid can also act as a signaling molecule to regulate gene expression in the liver [4].

AMP-activated protein kinase (AMPK) is a phylogenetically conserved serine/threonine protein kinase that has been proposed to act as a “metabolic master switch” that modulates hepatic lipid metabolism to adapt to environmental or nutritional stress factors [5], [6]. AMPK modulates hepatic lipid metabolism by regulating several lipid metabolism-related transcription factors such as peroxisome proliferator-activated receptor α (PPARα), sterol regulatory element-binding protein 1c (SREBP-1c), and carbohydrate responsive element-binding protein (ChREBP), all of which govern the expression of lipid metabolic enzymes [7]–[9]. Therefore, the AMPK signaling pathway plays a central role in hepatic lipid metabolism. AMPKα is activated in response to an increase in the ratio of AMP to ATP within the cell [10]. Furthermore, the liver kinase B1 (LKB1), a known tumor suppressor, is the upstream kinase in the AMP-activated protein kinase cascade [11]. Activation of AMPKα by LKB1 depends on the AMP/ATP ratio. Deleting LKB1 in the liver results in a proportional decrease in AMPKα phosphorylation at Thr172 [12]. Sirtuins1 (SIRT1) is also a fuel-sensing molecule that plays an important role in the regulation of cell energy metabolism. Study demonstrated that AMPK and SIRT1 regulated each other [13]. Price et al. [14] reported that resveratrol activated AMPK in a SIRT1-dependent manner through deacetylation of LKB1.

Kondo et al. [15] demonstrated that acetic acid-treated mice had lower triglyceride (TG) content in the liver and increased expression of lipid oxidation genes. Furthermore, Sakakibara et al. [16] reported that acetic acid activated hepatic AMPKα in diabetic KK-A(y) mice. Consequently, genes downstream of AMPKα that are involved in lipid oxidation were upregulated, increasing lipid oxidation. These studies demonstrated that acetic acid could reduce liver fat accumulation by activating the AMPK pathway. The hepatic lipid metabolism of dairy cows is different from that of monogastric animals such as humans and mice. And the effect of acetic acid on the hepatic lipid metabolism in dairy cows is also significantly different from that in humans and mice. However, in ruminants, it is unclear the mechanism of acetic acid on the regulation of hepatic lipid metabolism. Therefore, the objective of this study was to investigate the molecular mechanism by which acetic acid regulates lipid metabolism in bovine hepatocytes. The results of this study should provide insights into the physiological function of acetic acid in hepatic lipid metabolism in ruminants.

## Results

### Effect of the duration of acetic acid treatment on AMPKα phosphorylation and activity in hepatocytes

**Figure 1:**
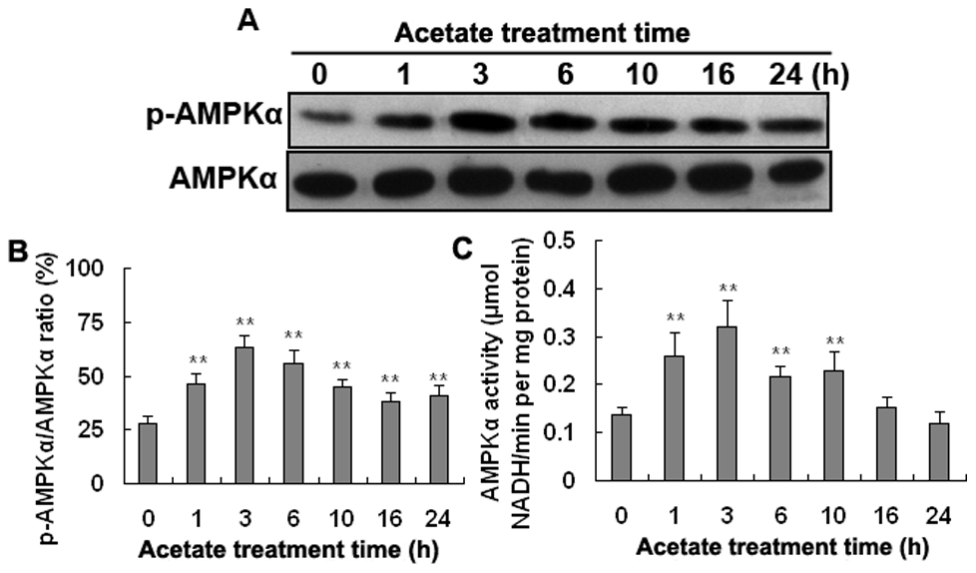
Effect of the duration of acetic acid treatment on AMPKα phosphorylation and activity in hepatocytes. Hepatocytes were treated with 3.6 mM acetate (neutralized acetic acid) for 0, 1, 3, 6, 10 16, and 24 h. A: Western blotting results of p-AMPKα and AMPKα. B: The phosphorylation level of AMPKα (p-AMPKα/AMPKα). C: AMPKα activity. * *p*<0.05, ** *p*<0.01 versus the control group.

The phosphorylation level of AMPKα (p-AMPKα/AMPKα) and AMPKα activity in hepatocytes were highest at 3 h of acetate treatment (Fig. 1A–1C; *p*<0.01). At 3 h, the phosphorylation level of AMPKα in the acetate-treated hepatocytes was 2.25-fold higher than in the control hepatocytes and AMPKα activity was 2.30-fold higher.

### Acetic acid activates AMPKα in hepatocytes

**Figure 2:**
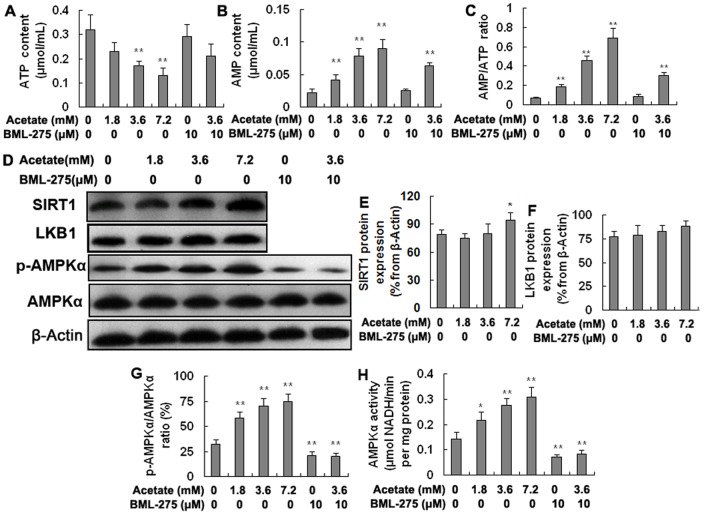
Acetic acid activates the AMPKα signaling pathway. Hepatocytes were treated with acetate and BML-275 and were divided into a control group (0 mM acetate), a low-dose acetate treatment group (1.8 mM acetate), a medium-dose acetate treatment group (3.6 mM acetate), a high-dose acetate treatment group (7.2 mM acetate), a BML-275 group (10 μM BML-275), and a BML-275+acetate group (10 μM BML-275+3.6 mM acetate). Acetate (sodium acetate) was used in the form of neutralized acetic acid to avoid changing the pH of the medium. A: AMP content. B: ATP content. C: AMP/ATP ratio. D: Western blotting results for SIRT1, LKB1, p-AMPKα, and AMPKα. E: The protein levels of SIRT1. F: The protein levels of LKB1. G: The phosphorylation level of AMPKα (p-AMPKα/AMPKα). H: AMPKα activity. * *p*<0.05, ** *p*<0.01 versus the control group.

To determine the effect of acetic acid on the AMPKα, we determined the AMP and ATP content, the AMPKα phosphorylation and activity, and the LKB1 protein expression. As shown in Figure 2, the ATP content was significantly lower in the medium- and high-dose treatment groups than in the control group (Fig. 2A; *p*<0.01). However, the AMP content was significantly increased in the acetate-treated groups (Fig. 2B; *p*<0.01). The AMP/ATP ratio increased from 2.00- to 10.00-fold from the low-dose acetate treatment group to the high-dose group (Fig. 2C; *p*<0.01). The phosphorylation level of AMPKα and AMPKα activity was also significantly higher in the acetate-treated groups than in the control group and was significantly lower in the BML-275 (an AMPKα inhibitor) and BML-275+acetate groups than in the control group (Fig. 2G, 2H; *p*<0.05 and *p*<0.01). Overall, these results demonstrate that acetic acid converts to acetyl-CoA with the consumption of ATP, resulting in a significant increase in the AMP/ATP ratio, which induces an increase of AMPKα phosphorylation and activity. The protein levels of SIRT1 were significantly higher in the high-dose acetate treatment group than in the control group (Fig. 2E; *p*<0.05). However, there was no significant change in the protein levels of LKB1 (Fig. 2F). SIRT1 activates AMPKα dependent on LKB1. These results indicate that high levels of acetic acid increase SIRT1 expression. However, acetic acid does not significantly affect the protein expression of LKB1.

### The expression and transcriptional activity of PPARα, SREBP-1c, and ChREBP in hepatocytes

**Figure 3:**
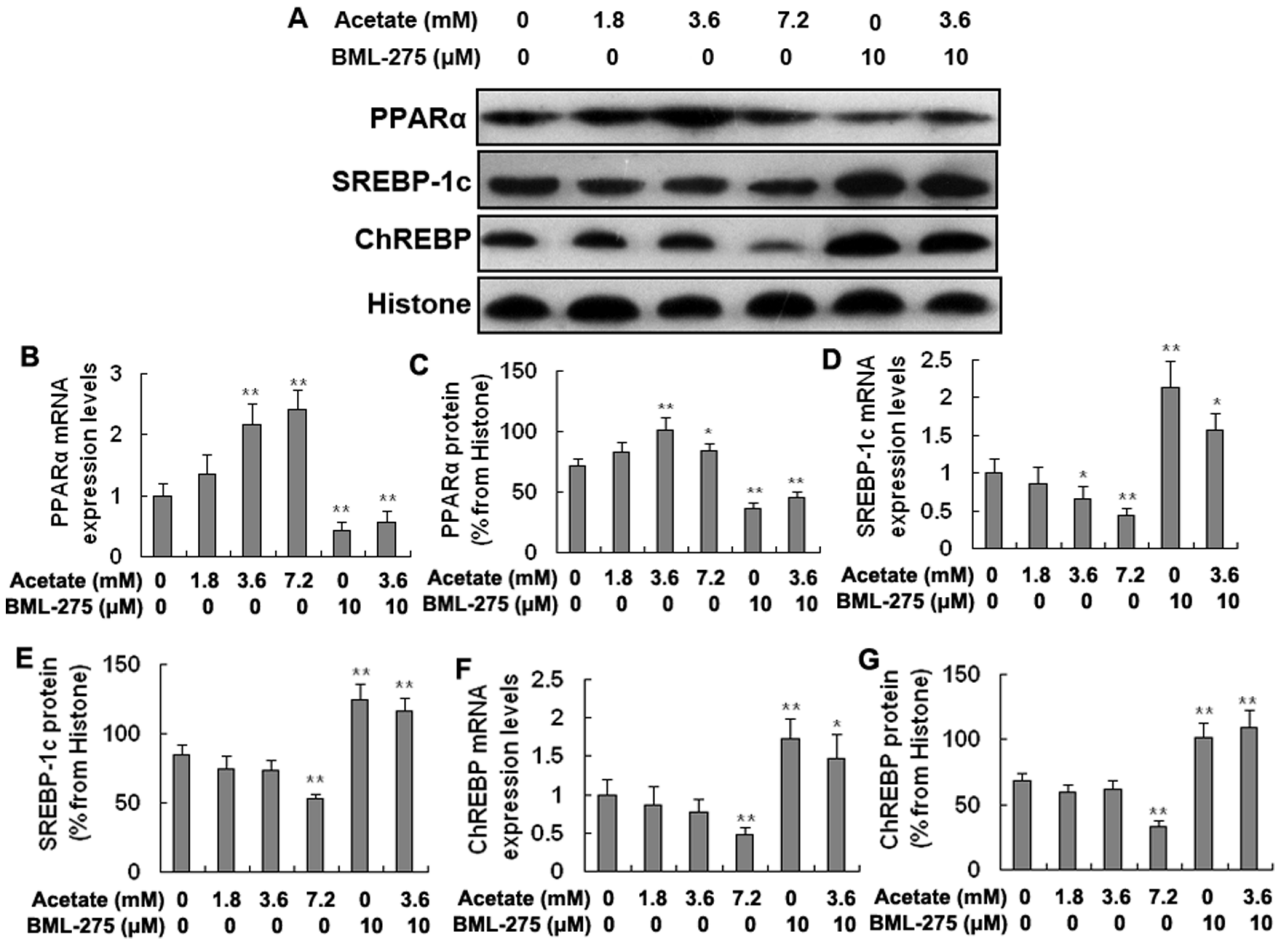
The mRNA and protein expression levels of PPARα, SREBP-1c, and ChREBP in the hepatocytes. Hepatocytes were treated with acetate and BML-275 and divided into a control group (0 mM acetate), a low-dose acetate treatment group (1.8 mM acetate), a medium-dose acetate treatment group (3.6 mM acetate), a high-dose acetate treatment group (7.2 mM acetate), a BML-275 group (10 μM BML-275), and a BML-275+acetate group (10 μM BML-275+3.6 mM acetate). Acetate (sodium acetate) was used in the form of neutralized acetic acid to avoid changing the pH of the medium. A: Western blotting results of PPARα, SREBP-1c, and ChREBP. B and C: mRNA and protein levels of PPARα. D and E: mRNA and protein levels of SREBP-1c. F and G: mRNA and protein levels of ChREBP. * *p*<0.05, ** *p*<0.01 versus the control group.

The mRNA and protein expression levels of PPARα were significantly higher in the medium- and high-dose acetate treatment groups than in the control group and were significantly lower in the BML-275 and BML-275+acetate groups than in the control group (Fig. 3B, 3C; *p*<0.05 and *p*<0.01). However, the SREBP-1c and ChREBP showed the opposite results. The SREBP-1c mRNA levels were significantly lower in the medium- and high-dose treatment groups than in the control group (Fig. 3D; *p*<0.05 and *p*<0.01), and the SREBP-1c protein levels were significantly lower in the high-dose treatment group (Fig. 3E; *p*<0.01). The SREBP-1c mRNA and protein levels were significantly higher in the BML-275 and BML-275+acetate groups than in the control group (Fig. 3D, 3E; *p*<0.05 and *p*<0.01). The mRNA and protein levels of ChREBP were significantly lower in the high-dose treatment group than in the control group and were significantly higher in the BML-275 and BML-275+acetate groups than in the control group (Fig. 3F, 3G; *p*<0.05 and *p*<0.01). Taken together, these findings suggest that acetic acid increases the expression of PPARα and decreases the expression of SREBP-1c and ChREBP in bovine hepatocytes.

**Figure 4:**
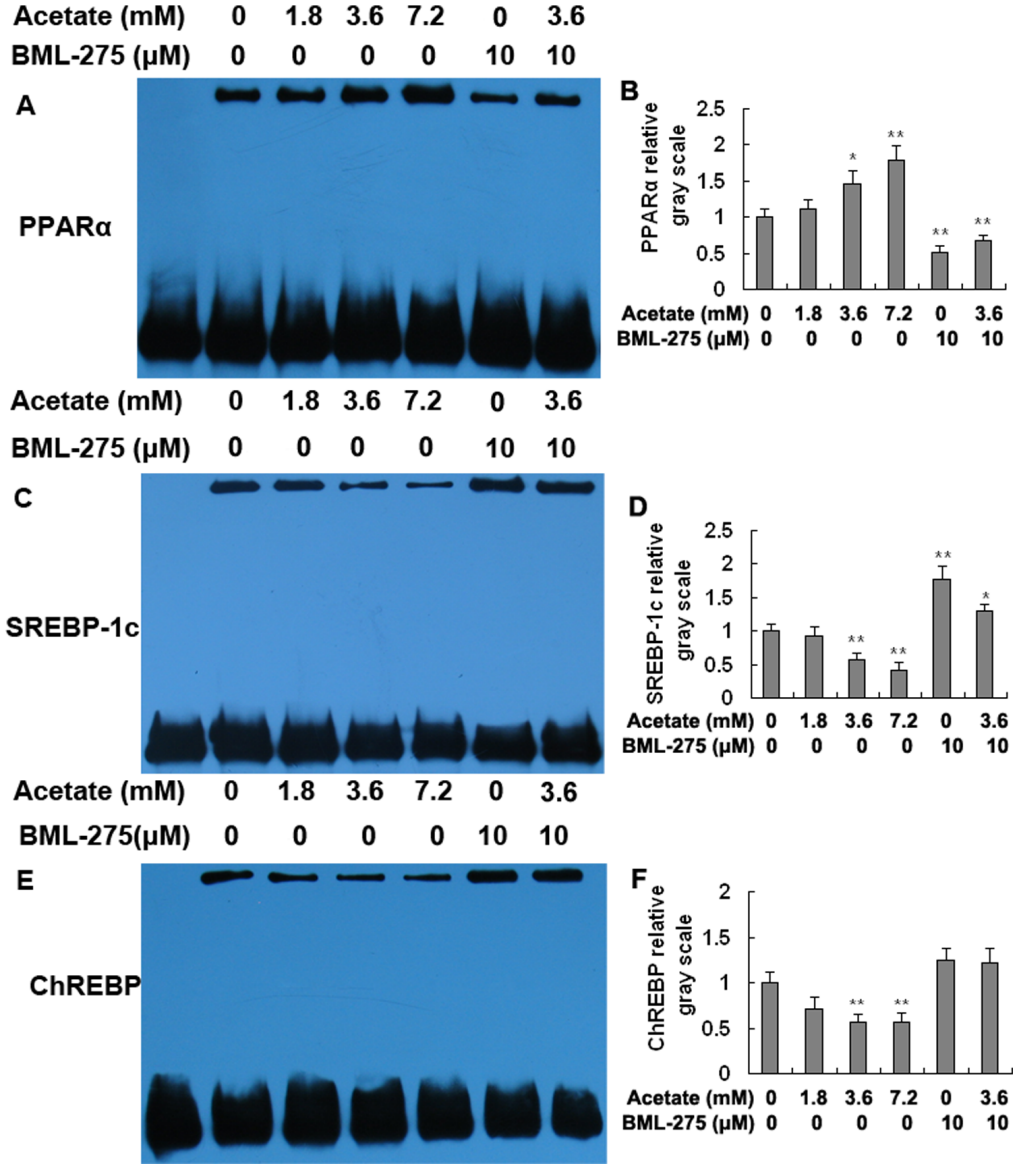
Transcriptional activity of PPARα, SREBP-1c, and ChREBP in hepatocytes. Hepatocytes were treated with acetate and BML-275 and divided into a control group (0 mM acetate), a low-dose acetate treatment group (1.8 mM acetate), a medium-dose acetate treatment group (3.6 mM acetate), a high-dose acetate treatment group (7.2 mM acetate), a BML-275 group (10 μM BML-275), and a BML-275+acetate group (10 μM BML-275+3.6 mM acetate). Acetate (sodium acetate) was used in the form of neutralized acetic acid to avoid changing the pH of the medium. A and B: EMSA results for PPARα. C and D: EMSA results for SREBP-1c. E and F: EMSA results for ChREBP. * *p*<0.05, ** *p*<0.01 versus the control group.

The transcriptional activity of PPARα was significantly higher in the medium- and high-dose acetate treatment groups than in the control group and was significantly lower in the BML-275 and BML-275+acetate groups than in the control group (Fig. 4A, 4B; *p*<0.05 and *p*<0.01). The transcriptional activity of SREBP-1c and ChREBP displayed the opposite trend. The transcriptional activity of SREBP-1c was significantly lower in the medium- and high-dose groups than in the control group and was markedly higher in the BML-275 and BML-275+acetate groups than in the control group (Fig. 4C, 4D; *p*<0.05 and *p*<0.01). The transcriptional activity of ChREBP was significantly lower in the medium- and high-dose groups (Fig. 4E, 4F; *p*<0.01). Taken together with the data presented in Fig. 4, these results strongly suggest that acetic acid increases the transcriptional activity of PPARα and inhibits the transcriptional activity of SREBP-1c and ChREBP in bovine hapatocytes.

### The mRNA expression levels of the PPARα, SREBP-1c, and ChREBP target genes in hepatocytes

**Figure 5:**
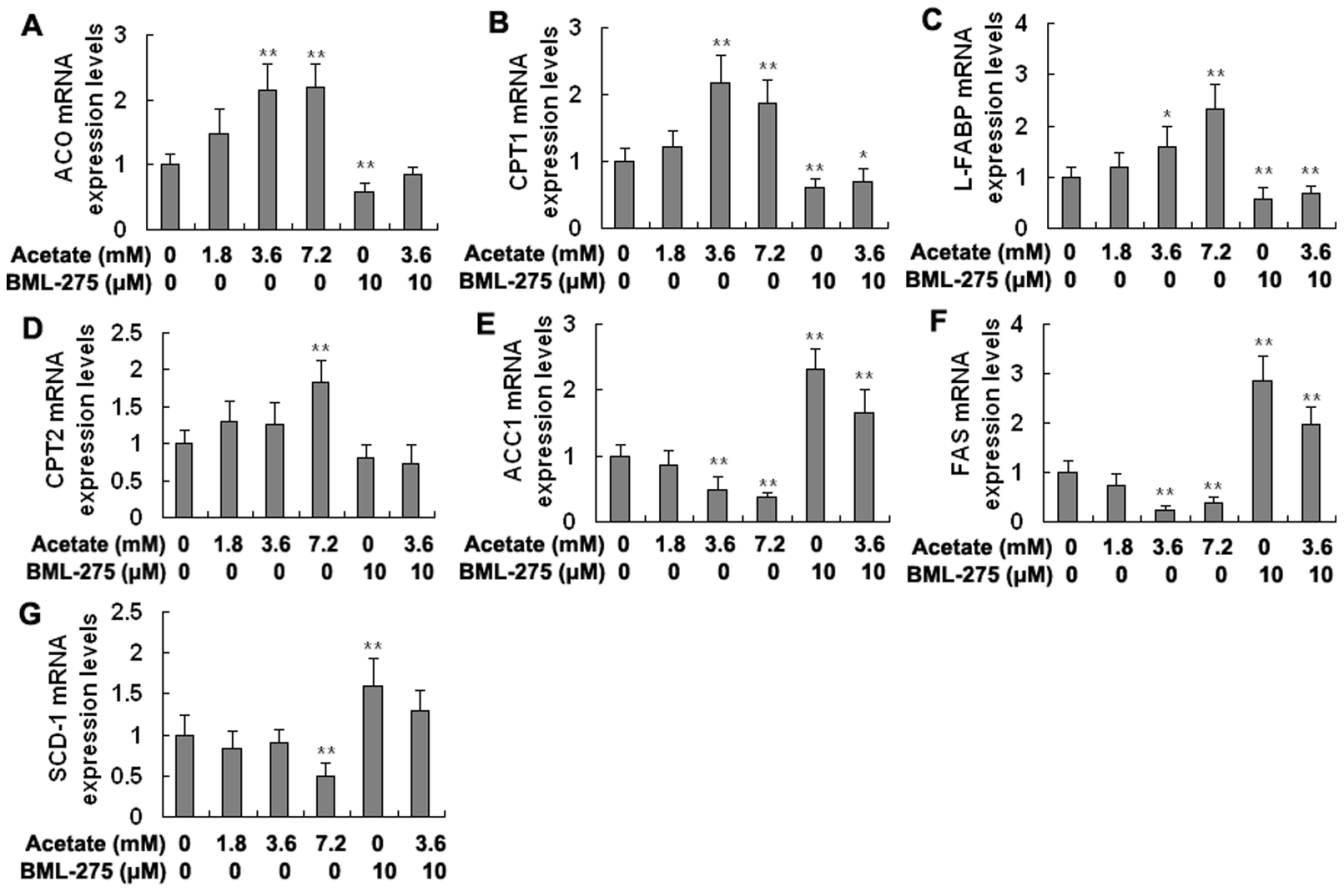
The mRNA expression levels of PPARα, SREBP-1c, and ChREBP target genes in hepatocytes. Hepatocytes were treated with acetate and BML-275 and divided into a control group (0 mM acetate), a low-dose acetate treatment group (1.8 mM acetate), a medium-dose acetate treatment group (3.6 mM acetate), a high-dose acetate treatment group (7.2 mM acetate), a BML-275 group (10 μM BML-275), and a BML-275+acetate group (10 μM BML-275+3.6 mM acetate). Acetate (sodium acetate) was used in the form of neutralized acetic acid to avoid changing the pH of the medium. A-D: mRNA expression levels of ACO, CPT1, L-FABP, and CPT2, respectively. E-G: mRNA expression levels of ACC1, FAS, and SCD-1, respectively. * *p*<0.05, ** *p*<0.01 versus the control group.

The mRNA levels of the PPARα target genes acyl-CoA oxidase (ACO), carnitine palmitoyltransferase 1 (CPT1), carnitine palmitoyltransferase 2 (CPT2), and liver fatty acid-binding protein (L-FABP) tended to increase in the acetate-treated groups. The mRNA levels of ACO, CPT1, and L-FABP were markedly higher in the medium- and high-dose acetate treatment groups than in the control group (Fig. 5A-5C; *p*<0.05 and *p*<0.01). The mRNA levels of CPT2 were significantly higher in the high-dose group than in the control group (Fig. 5D; *p*<0.01). However, the mRNA levels of ACO, CPT1, and L-FABP were significantly lower in the BML-275 and BML-275+acetate groups than in the control group (Fig. 5A-5C; *p*<0.05 and *p*<0.01). Overall, these results demonstrate that acetic acid upregulates the mRNA expression of lipid oxidation genes by increasing the expression and transcriptional activity of PPARα in bovine hepatocytes.

In contrast, the mRNA expression levels of the SREBP-1c and ChREBP target genes, including acetyl-CoA carboxylase 1 (ACC1), fatty acid synthase (FAS), and stearoyl-CoA desaturase-1 (SCD-1), tended to decrease in the acetate-treated groups. The mRNA levels of ACC1 and FAS were significantly lower in the medium- and high-dose groups than in the control group and were significantly higher in the BML-275 and BML-275+acetate groups than in the control group (Fig. 5E, 5F; *p*<0.01). The mRNA levels of SCD-1 were significantly lower in the high-dose group and were markedly higher in the BML-275 group than in the control group (Fig. 5G; *p*<0.01). Collectively, these results suggest that acetic acid inhibits the mRNA expression of lipid synthesis genes through inhibiting the expression and transcriptional activity of SREBP-1c and ChREBP in bovine hepatocytes.

### The phosphorylation level and enzyme activity of ACC1 in hepatocytes

**Figure 6:**
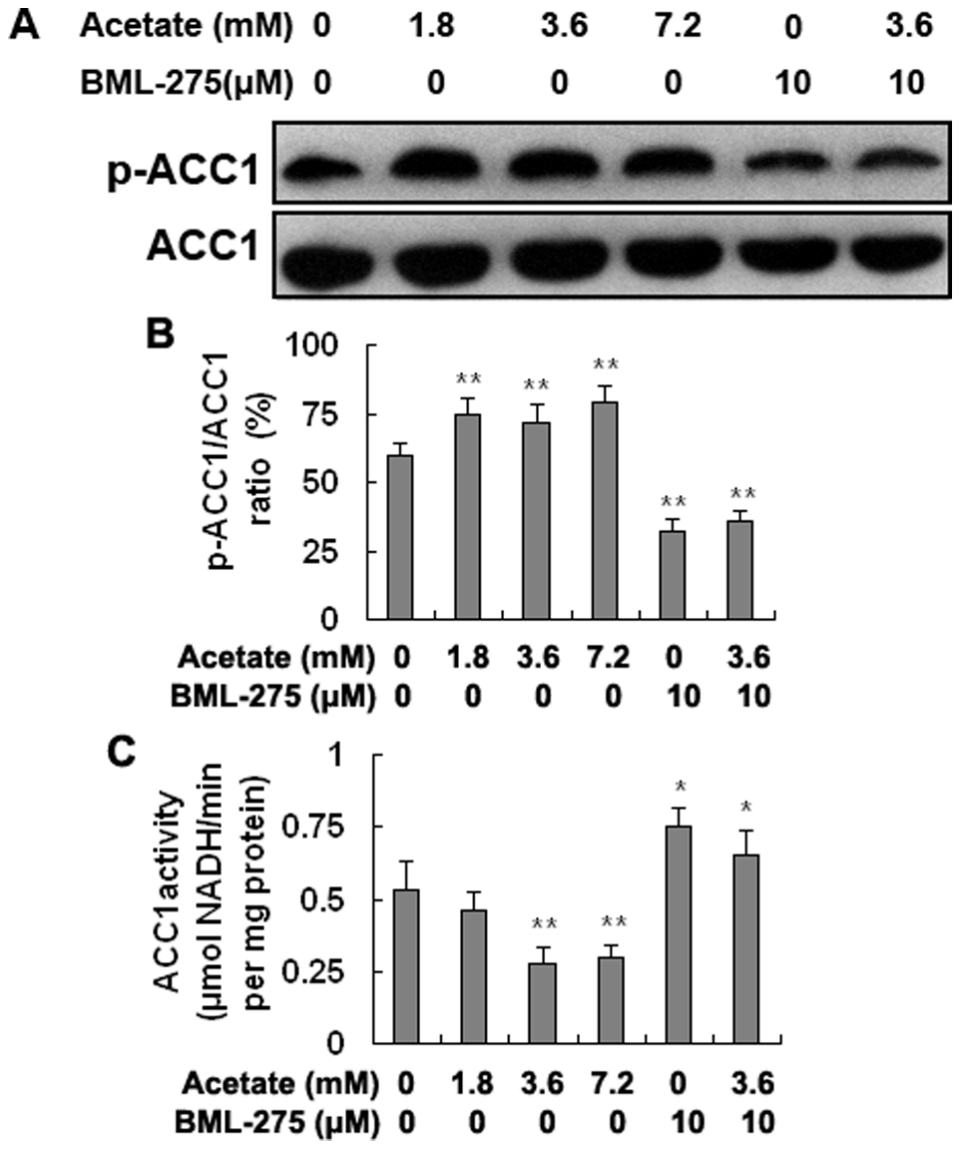
The phosphorylation level and enzyme activity of ACC1. Hepatocytes were treated with acetate and BML-275 and divided into a control group (0 mM acetate), a low-dose acetate treatment group (1.8 mM acetate), a medium-dose acetate treatment group (3.6 mM acetate), a high-dose acetate treatment group (7.2 mM acetate), a BML-275 group (10 μM BML-275), and a BML-275+acetate group (10 μM BML-275+3.6 mM acetate). Acetate (sodium acetate) was used in the form of neutralized acetic acid to avoid changing the pH of the medium. A: Western blotting results for p-ACC1 and ACC1. B: The phosphorylation level of ACC1. C: Enzyme activity of ACC1. * *p*<0.05, ** *p*<0.01 versus the control group.

The phosphorylation level of ACC1 (p-ACC1/ACC1) increased in an acetate dose-dependent manner and was significantly higher in the acetate-treated groups than in the control group. It was significantly lower in the BML-275 and BML-275+acetate groups than in the control group (Fig. 6B; *p*<0.01). ACC1 activity was significantly lower in the medium- and high-dose groups than in the control group and was markedly higher in the BML-275 and BML-275+acetate groups than in the control group (Fig. 6C; *p*<0.01 and *p*<0.05). These results indicate that acetate-activated AMPKα can directly phosphorylate ACC1 and inhibit its activity, which inhibits lipid synthesis in bovine hepatocytes.

### Triglycerides content

**Figure 7:**
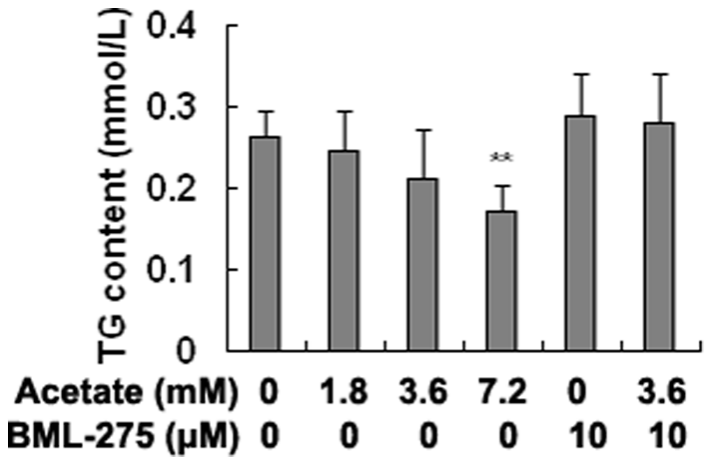
TG content in hepatocytes. Hepatocytes were treated with acetate and BML-275 and divided into a control group (0 mM acetate), a low-dose acetate treatment group (1.8 mM acetate), a medium-dose acetate treatment group (3.6 mM acetate), a high-dose acetate treatment group (7.2 mM acetate), a BML-275 group (10 μM BML-275), and a BML-275+acetate group (10 μM BML-275+3.6 mM acetate). Acetate (sodium acetate) was used in the form of neutralized acetic acid to avoid changing the pH of the medium. * *p*<0.05, ** *p*<0.01 versus the control group.

Acetate decreased hepatocyte TG content in a dose-dependent manner. The TG content was 35% lower in the high-dose acetate treatment group than in the control group (Fig. 7; *p*<0.01). Taken together, our *in vitro* data demonstrate that acetic acid activates AMPKα signaling pathway to reduce TG content in bovine hepatocytes.

## Discussion

In dairy cows, the liver is the main organ responsible for modulating lipid metabolism and maintaining lipid homeostasis through responses to nutrient signals [17]. In recent years, studies have shown that acetic acid can act as a signaling molecule that modulates the expression of lipid metabolism genes in hepatocytes [9], [18]. An *in vitro* HepG2 cell study demonstrated that acetic acid activates AMPKα, which in turn upregulates the expression of lipid oxidation genes in the liver to reduce fat accumulation [16]. A liver-specific AMPKα deletion in mice leads to increased plasma TG content and hepatic lipogenesis [19].

The blood acetate concentration is dozens of times higher in dairy cows than that in humans and mice. Furthermore, the biological function of acetic acid in ruminants is different from that in humans and mice. However, it is not clear whether acetic acid activates the AMPK signaling pathway in the ruminant liver. In this study, we observed that the AMP/ATP ratio increased 2.00- to 10.00-fold in acetate-treated hepatocytes. The phosphorylation level of AMPKα and AMPKα activity were significantly increased in acetate-treated groups and were significantly lower in the BML-275 and BML-275+acetate groups than in the control group. Abound acetate was converted to acetyl-CoA with the consumption of ATP in hepatocytes, resulting in a significant increase in the AMP/ATP ratio. The high AMP/ATP ratio increased AMPKα phosphorylation with the help of LKB1. These results demonstrate that acetic acid activates AMPKα in bovine hepatocytes. SIRT1 and AMPK is the cell metabolism regulator that regulates the cell energy metabolism [13]. An *in vivo* study demonstrated that SIRT1 activated AMPK dependent on the LKB1 [20]. In this study, the protein levels of SIRT1 were significantly increased in the high-dose acetate treatment group. However, there was no significant change in the protein levels of LKB1. These results suggest that acetic acid does not significantly affect the SIRT1 and LKB1 in bovine hepatocytes, which may be due to the difference of energy metabolism in the bovine hepatocytes. The hepatic energy metabolism of dairy cows is different from that of monogastric animals such as humans and mice. Taken together, these results indicate that acetic acid activates AMPK signaling pathway mainly through consumption of the intracellular ATP.

AMPK acts as a key metabolic “masters witch” by regulating target transcription factors involved in lipid metabolism, including PPARα, SREBP-1c and ChREBP. PPARα is a ligand-activated transcription factor that plays a key role in the regulation of the expression of lipid oxidation genes, including ACO, CPT1, L-FABP, and CPT2 [21], [22]. ACO is a rate-limiting enzyme in fatty acid β oxidation [23]. CPT1 and CPT2 transfer long-chain acyl-CoA into the mitochondria for β oxidation [20]. L-FABP regulates the intake and transport of fatty acids in the cell [24]. ACO, CPT1, L-FABP, and CPT2, which are regulated by PPARα, are the key enzymes of lipid oxidation in hepatocytes. In an *in vitro* study, HepG2 cells treated with 100, 200, or 500 μM acetate displayed significantly increased expression of PPARα and its target genes, including ACO and CPT1 [15]. However, in mice treated with acetic acid, Sakakibara et al. [16] demonstrated that acetic acid did not affect the transcription of PPARα and that ACO mRNA levels were not significantly increased.

In this study, we demonstrated that acetic acid could activate AMPKα. The expression levels and transcriptional activity of PPARα were significantly increased in the medium- and high-dose acetate treatment groups and were significantly lower in the BML-275 and BML-275+acetate groups than in the control group. These results indicate that acetic acid-activated AMPKα promotes the expression and transcriptional activity of PPARα. Moreover, the mRNA expression levels of PPARα target genes, including ACO, CPT1, L-FABP, and CPT2, were significantly upregulated in the acetate-treated groups. Acetic acid activates PPARα, which increases the expression of lipid oxidation genes, thereby increasing lipolysis in bovine hepatocytes. The blood concentration of acetic acid is dozens of times higher in dairy cows than in mice, and this high concentration of acetic acid promotes lipolysis in the hepatocytes of dairy cows. The variations in the effect of acetic acid on lipolysis may be due to differences in the treatment concentrations of acetate and animal species among experiments.

SREBP-1c and ChREBP govern lipogenesis through the transcriptional regulation of lipogenic genes, including ACC1, FAS, and SCD-1 [25], [26]. The synthesis of malonyl-CoA is the first committed step of fatty acid synthesis, and the enzyme that catalyzes this reaction, ACC1, is the major regulatory site in fatty acids synthesis [27]. FAS and SCD-1 catalyze fatty acid elongation and desaturation steps, respectively. FAS is a determinant of the maximal capacity of the liver to synthesize fatty acids by de novo lipogenesis [28]. SCD1 catalyzes the synthesis of monounsaturated fatty acids, particularly oleate (C18:1n-9) and palmitoleate (C16:1n-7), which are the major components of TG [28]. ACC1, FAS, and SCD-1 are the key rate-controlling enzymes in lipid synthesis. The DNA-binding activity of ChREBP is significantly decreased in the livers of rats fed a high fat diet [29]. Sakakibara et al. [16] reported that SREBP-1c mRNA expression levels were significantly decreased in rat primary hepatocytes treated with 200 μM acetate. Furthermore, an *in vivo* study demonstrated that the administration of acetic acid to rats decreased the expression of lipogenic genes such as ACC1 and FAS [30]. However, Kondo et al. [15] did not observe changes in the expression of SREBP-1c and its target genes in acetic acid-treated rats. Taken together, these conflicting results demonstrate that the effect of acetic acid on the expression and transcriptional activity of SREBP-1c and ChREBP is not well understood, particularly in ruminants.

In this study, we demonstrated that the expression and transcriptional activity of SREBP-1c and ChREBP were significantly decreased in the acetate-treated groups but were markedly increased in the BML-275 and BML-275+acetate groups. These results indicate that acetic acid inhibits the expression and transcriptional activity of SREBP-1c and ChREBP. Furthermore, the mRNA expression levels of the SREBP-1c and ChREBP target genes ACC1, FAS, and SCD-1 were significantly lower in the acetate-treated groups than in the control group. Taken together, these results indicate that acetic acid inhibits the expression and transcriptional activity of SREBP-1c and ChREBP, thereby down-regulating the expression of lipid synthesis genes and decreasing lipid synthesis in bovine hepatocytes. The downregulation of ChREBP and SREBP-1c together provides a molecular explanation for the well-known shift in hepatic lipid metabolism from lipid synthesis and storage to oxidation associated with high acetic acid.

Studies have shown that activated AMPKα can directly phosphorylate and inactivate ACC1 [31]. In this study, the phosphorylation level of ACC1 was significantly increased in the acetate-treated groups. However, ACC1 activity was markedly decreased as phosphorylation increased. These results indicate that acetic acid-activated AMPKα increases the phosphorylation level of ACC1and inhibits its enzyme activity in bovine hepatocytes, thereby blocking fatty acid synthesis.

Acetic acid acts as a signaling molecule and activates the AMPK signaling pathway, thereby increasing lipolysis and decreasing lipid synthesis in bovine hepatocytes. Consequently, the TG content was significantly decreased in acetate-treated hepatocytes, reducing fat accumulation in the liver. The ability of the TG export in the liver of dairy cows is lower than that of humans or rodents [32], resulting in a higher incidence of fatty liver in dairy cows than in monogastric animals. The ability of acetic acid to activate the AMPK signaling pathway to reduce fat accumulation in the liver could be exploited to prevent fatty liver in ruminants.

**Figure 8:**
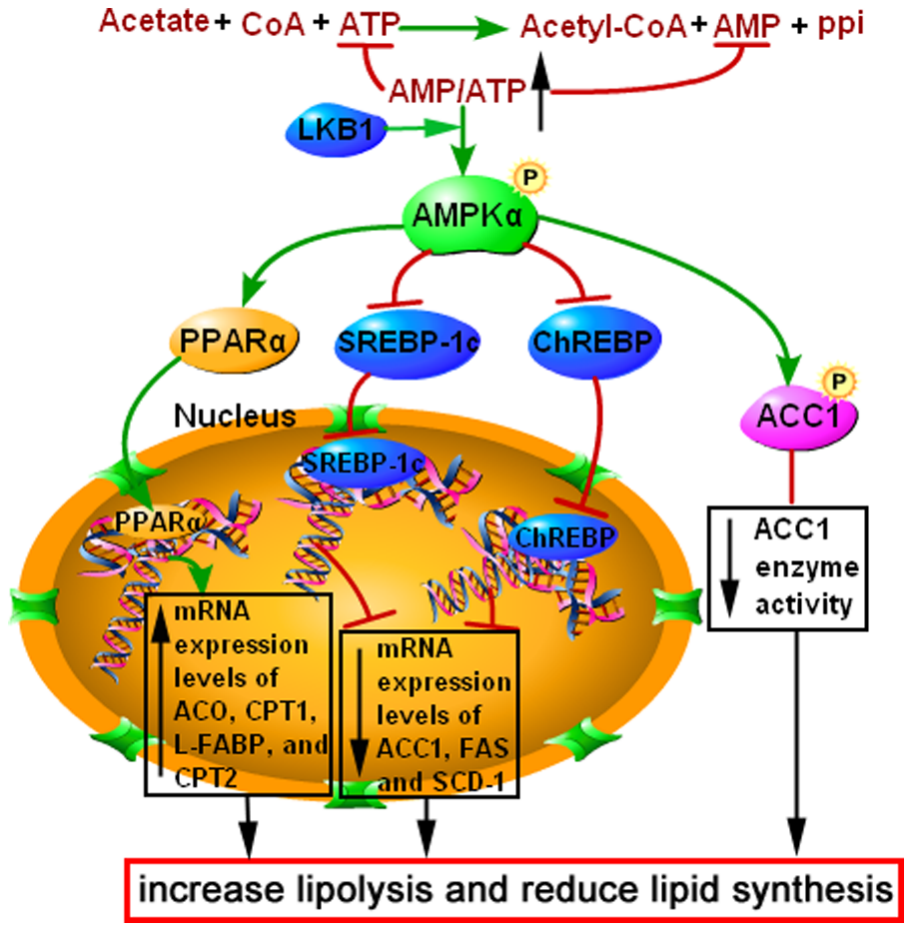
Acetic acid activates the AMP-activated protein kinase signaling pathway to regulate lipid metabolism in bovine hepatocytes. Acetic acid is metabolized to acetyl-CoA with consumption of ATP in bovine hepatocytes, resulting in a significant elevation of the AMP/ATP ratio and the phosphorylation and activation of AMPKα. Activated AMPKα increases the expression and transcriptional activity of PPARα, thereby increasing the expression of lipolytic genes, including ACO, CPT1, CPT2, and L-FABP. AMPKα activation inhibits the expression and transcriptional activity of SREBP-1c and ChREBP, thereby reducing the expression of lipogenic genes, including ACC1, FAS, and SCD-1. In addition, activated AMPKα directly phosphorylates and inhibits ACC1. Consequently, acetic acid increases lipolysis and reduces lipid synthesis in bovine hepatocytes.

In conclusion, the current study indicates that acetic acid can act as a signaling molecule to significantly increase lipolysis and decrease lipid synthesis in bovine hepatocytes. A potential mechanism in which acetic acid is metabolized to acetyl-CoA in hepatocytes, with the consumption of ATP, is shown in Figure 8. An elevated AMP/ATP ratio increases the phosphorylation and activity of AMPKα. Activated AMPKα promotes the expression and transcriptional activity of PPARα, thereby increasing the expression of lipolytic genes. Furthermore, activated AMPKα inhibits the expression and transcriptional activity of SREBP-1c and ChREBP, thereby reducing the expression of lipogenic genes. In addition, activated AMPKα directly phosphorylate and inhibit ACC1. Consequently, acetic acid increases lipolysis and decreases lipid synthesis in bovine hepatocytes, which reduces hepatic fat accumulation in dairy cows. The current study identifies a biochemical mechanism for the regulation of hepatic lipid metabolism by acetic acid in dairy cows.

## Materials and Methods

### Materials

Fetal bovine serum, collagenase IV, HepatoZYME medium and RPMI-1640 medium were purchased from Gibco (Grand Island, NY, USA). Insulin and HEPES were obtained from Sigma-Aldrich (Saint Louis, MO, USA). Dexamethasone acetate, vitamin C, ascorbic acid, penicillin, streptomycin, and other chemicals were obtained from Baoman Biotechnology (Shanghai, China). BML-275 (an AMPKα inhibitor) was purchased from Santa Cruz Biotechnology, Inc. (Santa Cruz, CA, USA).

### Cell culture

Hepatocytes were isolated using the collagenase perfusion method and were obtained as previously described [33], [34]. The study protocol was approved by the Ethics Committee on the Use and Care of Animals, Jilin University (Changchun, China). Briefly, the caudate lobe of the liver was obtained through surgical excision from a female Holstein calf anesthetized with thiamylal sodium under sterile conditions. The liver was perfused with perfusion solution to wash away the blood until the perfusion solution became clear. The liver was then perfused with a collagenase IV solution to digest the liver tissue. After digestion, the liver capsule was cut off. A total of 100 mL basic medium containing 0.2% bovine serum albumin was added to terminate digestion. The liver capsule, blood vessels, fat, and any parts of the liver caudate lobe that were incompletely digested were removed. The hepatocyte suspension was filtered sequentially with 100 mesh (150 µm), 200 mesh (75 µm), and 500 mesh (30 µm) cell sieves. Then, the hepatocyte suspension was washed twice with basic medium. The cell density was adjusted to 1×10^6^ cells/mL with adherent culture medium. The hepatocyte suspension was seeded into a 6-well tissue culture plate (2 mL per well) and incubated at 37°C in 5% CO_2_. After a 4-h attachment period, the medium was replaced with growth medium containing 10% fetal bovine serum. The medium was replaced with fresh medium every 24 h.

### Acetic acid treatment

Cells were serum-starved overnight and then treated with sodium acetate (acetate) in the form of neutralized acetic acid to avoid changing the pH of the medium. The concentration of acetate was chosen according to the normal hematology standards of dairy cows. The hepatocytes were subjected to the following treatments. For time course experiments, hepatocytes were treated with 3.6 mM acetate for 0, 1, 3, 6, 10, 16, and 24 h. For dose-response experiments, hepatocytes were treated with acetate and BML-275 (BML-275 is an AMPKα inhibitor that inhibits AMPKα phosphorylation at threonine-172 [35]). The treated hepatocytes were divided into a control group (0 mM acetate), a low-dose acetate treatment group (1.8 mM acetate), a medium-dose acetate treatment group (3.6 mM acetate), a high-dose acetate treatment group (7.2 mM acetate), a BML-275 group (10 μM BML-275), and a BML-275+acetate group (10 μM BML-275+3.6 mM acetate); all groups were treated for 3 h. Each treatment concentration of acetate or BML-275 was replicated 24 times.

### AMP and ATP level determination

Hepatocytes were harvested with a cell scraper and collected directly into 0.5 mL ice-cold 6% (v/v) perchloric acid. The cells were lysed by pipetting up and down repeatedly. The lysate was centrifuged for 5 min at 12000×*g* at 4°C. The supernatant was used to determine the content of AMP and ATP by reverse-phase HPLC analysis (Thermo Fisher Scientific, Waltham, MA, USA).

### AMPKα and ACC1 activity determination

Hepatocytes were harvested with a cell scraper and transferred into a centrifuge tube. The cells were washed twice with ice-cold PBS and lysed with lysis buffer (Shanghai Bluegene Biotech Co., Ltd., Shanghai, China) in an ice bath for 30 min. The lysate was then centrifuged for 5 min at 12000×*g* at 4°C, and the supernatant was used to determine the enzyme activity of AMPKα and ACC1 using a biochemical kit (Shanghai Bluegene Biotech Co., Ltd.) according to the supplier's protocol.

### Triglyceride content determination

Hepatocytes were harvested with a cell scraper and transferred into a centrifuge tube. The cells were washed twice with ice-cold PBS and lysed with an SL-1000D ultrasonic cell disruption apparatus (Shunliu Instrument Company, Nanjing, China). The lysate was centrifuged for 5 min at 12000×*g* at 4°C, and the supernatant was used to determine the triglyceride content using an automatic biochemical analyzer (Shenyang EKSV Medical Equipment Co., Ltd., China).

### RNA extraction and real-time RT-PCR

Hepatocyte RNA was extracted with a Takara RNA extraction kit (Takara Biotechnology Co., Ltd., Dalian, China) according to the manufacturer's instructions. RNA integrity was determined by electrophoresis on 1% agarose gels. The RNA was analyzed by spectrophotometry at 260 and 280 nm using a Gene Quant II RNA/DNA Calculator (Pharmacia Biotech, Cambridge, UK). Samples with an optical density ratio of RNA at 260/280 nm >1.8 were used for further analyses. The RNA was transcribed into cDNA using a reverse transcription kit (Takara Biotechnology Co., Ltd.) according to the manufacturer's protocol. Gene primers were designed using Primer Express software (PE Applied Biosystems, Inc., Foster City, CA, USA) and shown in Table 1. The mRNA expression levels were evaluated by quantitative polymerase chain reaction (qRT-PCR) analysis using a SYBR Green QuantiTect RT-PCR Kit (Takara Biotechnology Co., Ltd.). qRT-PCR was performed on a 7500 Real-Time PCR System (Applied Biosystems). The relative expression of each gene was normalized to β-actin levels.

**Table 1:** The primers sequences of the genes.

Gene	Sequence number	Primer sequences (5′-3′)	Length
PPARα	XM_341399	For TCAGATGGCTCCGTTATT	132 bp
		Rev CCCGCAGATCCTACACT	
SREBP-1c	NM_001113302.1	For GCAGCCCATTCATCAGCCAGACC	119bp
		Rev CGACACCACCAGCATCAACCACG	
ChREBP	NM_001205408.1	For ATCCGCCTCAACAACGC	144bp
		Rev TCCCTCCAAGACGACG	
ACO	NC_015500.1	For TAAGCCTTTGCCAGGTATT	189bp
		Rev ATGGTCCCGTAGGTCAG	
CPT1	NC_007330.4	For GGTCAACAGCAACTACTACG	188bp
		Rev TGAACATCCTCTCCATCTGG	
CPT2	NM_001045889.1	For ACGCCGTGAAGTATAACCCT	119bp
		Rev CCAAAAATCGCTTGTCCCTT	
L-FABP	NC_007309.4	For AAGTACCAAGTCCAGACCCAG	111bp
		Rev CACGATTTCCGACACCC	
ACC1	NM_174224.2	For TCCTGCTGCTATTGCTACTCCA	95bp
		Rev CAGTCCCCGCACTCACATAA	
SCD-1	NM_173959.4	For GGCACATCAACTTTACCACG	136bp
		Rev CAGCCACTCTTGTAGCTTTCCTC	
FAS	NM_001012669.1	For ACAGCCTCTTCCTGTTTGACG	144bp
		Rev CTCTGCACGATCAGCTCGAC	
β-actin	BC142413.1	For GCCCTGAGGCTCTCTTCCA	101bp
		Rev GCGGATGTCGACGTCACA	

### Western blotting

Hepatocytes were harvested and washed twice in ice-cold PBS. Total cellular proteins and nuclear proteins were extracted using a protein extraction kit and a nuclear protein extraction kit (Sangon Biotech Co., Ltd., Shanghai, China) according to the manufacturer's instructions. Protein concentrations were measured with the Bio-Rad protein assay reagent (Bio-Rad, München, Germany). Proteins were separated on polyacrylamide gels and electrotransferred onto PVDF membranes. The membranes were blocked in bovine serum albumin/TBST buffer for 4 h and hybridized with antibodies specific for SIRT1, LKB1, AMPKα, phosphorylated AMPKα (p-AMPKα), ACC1, phosphorylated ACC1 (p-ACC1), PPARα, SREBP-1c, and ChREBP (Cell Signaling Technology, Inc., Danvers, MA, USA; Santa Cruz Biotechnology) overnight at 4°C. The membranes were then incubated with the appropriate peroxidase-conjugated secondary antibodies. Immunoreactive bands were detected with an enhanced chemiluminescence solution (ECL, Beyotime Biotechnology Inc., China). The blots were exposed to X-ray film, and the band intensity was measured using BandScan software version 5.0 (Glyco).

### Electrophoretic Mobility Shift Assay

An electrophoretic mobility shift assay (EMSA) was used to detect the transcriptional activity of PPARα, SREBP-1c, and ChREBP. Nuclear proteins were extracted using a nuclear protein extraction kit (Sangon Biotech Co., Ltd, Shanghai, China) according to the manufacturer's instructions. Protein concentrations were measured with the Bio-Rad protein assay reagent (Bio-Rad, Munich, Germany). The special probe recognition sequences for PPARα, SREBP-1c, and ChREBP are shown in Table 2. The probes were labeled with biotin for 30 min at 37°C. The binding reaction was performed according to the manufacturer's protocol using the Lightshift EMSA Optimization and Control Kit (Pierce Biotechnology, Inc., Rockford, IL, USA) and 4 μg of nuclear extract protein for 20 min at room temperature. DNA-protein complexes were separated by electrophoresis on non-denaturing 6.5% polyacrylamide TBE gels and were electrotransferred onto a nylon membrane, which was UV crosslinked (Cany Precision Instruments Co., Ltd., Shanghai, China). The biotin-labeled probe was detected with a chemiluminescence solution (Pierce Biotechnology, Inc.). The blots were exposed to X-ray film, and the band intensity was measured using BandScan software version 5.0 (Glyco).

**Table 2:** Probe sequences of PPARα, SREBP-1c, and ChREBP used in the electrophoretic mobility shift assay.

Transcription Factor	Sequence (5′-3′)
PPARα	CAAAACTAGGTCAAAGGTCA
SREBP-1c	GGAGGCATCACCCCACCGAC
ChREBP	TCCTGCATGTGCCACAGGCGTGTCACC

### Statistical analysis

Results are expressed as the mean ± standard deviation (SD). SPSS (Statistical Package for the Social Sciences) 16.0 software (SPSS Incorporated, Chicago, IL, USA) was used to analyze the data. The group differences were compared using Duncan's multiple range test. A *p* value of less than 0.05 was considered statistically significant, and values less than 0.01 were considered markedly significant.
